# Nurses’ Perception of the Effect of Motivation, Psycho-Social Safety Climate, and Work Engagement: A Mediation Analysis

**DOI:** 10.3390/nursrep15110376

**Published:** 2025-10-24

**Authors:** Eman Kamel Hossny, Nahed Shawkat Aboelmagd, Shimaa Elwardany Aly, Manal Mohamed Abd Elnaeem, Naglaa Saad Abd El-aty, Aml Moubark Mahmoud, Intisar Alsheikh Mohamed, Asmaa Mohamed Ahmed

**Affiliations:** 1 Community and Mental Health Nursing Department, Faculty of Nursing, Zarqa University, Zarqa 13132, Jordan; 2 Nursing Administration Department, Faculty of Nursing, Assiut University, Assiut 71515, Egypt; nahedshawkat@aun.edu.eg; 3 Primary Care Nursing Department, Faculty of Nursing, Al-Ahliyya Amman University, Amman 19328, Jordan; 10653@ammanu.edu.jo (S.E.A.); n.aty@ammanu.edu.jo (N.S.A.E.-a.); drasmaa@nurs.svu.edu.eg (A.M.A.); 4 Community Health Nursing, Faculty of Nursing, Assiut University, Assiut 71515, Egypt; 5 Critical Care & Emergency Nursing, Faculty of Nursing, Zarqa University, Zarqa 13132, Jordan; momar@zu.edu.jo; 6 Critical Care & Emergency Nursing, Faculty of Nursing, Assiut University, Assiut 71515, Egypt; 7 Business Administration Department, Higher Institute of E-Commerce Systems, Sohag 82524, Egypt; dr.aml.moubark@gmail.com; 8 Department of Nursing, College of Applied Medical Sciences, University of Jeddah, Jeddah 21589, Saudi Arabia; iaalsheikh@uj.edu.sa; 9 Nursing Administration Department, Faculty of Nursing, Qena University, Qena 83523, Egypt

**Keywords:** motivation, psychosocial safety climate, work engagement, nurses, mediation analysis

## Abstract

Nurses often work under high stress and heavy workloads, making it critical to understand factors that influence their motivation, psychosocial safety climate (PSC), and work engagement (WE). **Objectives**: To assess nurses’ levels of motivation, PSC, and WE; examine relationships among these variables; and test whether PSC mediates the association between motivation and WE. **Methods**: A descriptive correlational study was conducted with 318 nurses from Assiut University Hospital, Egypt, using validated scales for motivation, PSC, and WE. Data were analyzed using descriptive statistics, Pearson’s correlations, multivariate regression, and mediation analysis (bootstrapped, 5000 resamples). Statistical significance was set at *p* < 0.05. **Results**: Nurses reported moderate motivation (M = 118.1, SD = 16.4), moderate WE (M = 22.7, SD = 5.8), and low PSC perception (M = 60.5, SD = 16.6). Motivation was positively correlated with PSC (r = 0.48, 95% CI [0.39, 0.56], *p* < 0.001). Motivation-WE correlation was small and non-significant (r = 0.10, 95% CI [−0.01, 0.21], *p* = 0.08). Mediation analysis showed PSC partially mediated the motivation-WE link (indirect effect = 0.07, 95% CI [0.02, 0.14]), though the effect size was small. **Conclusions**: Motivation and PSC reinforce each other, but neither strongly predicts WE in this setting. Targeted strategies to strengthen PSC and intrinsic motivation may indirectly enhance engagement and retention.

## 1. Introduction

Nurses are the backbone of healthcare systems, providing direct patient care, coordinating multidisciplinary teams, and ensuring continuity of services. However, nursing is widely recognized as a high-stress profession, characterized by long shifts, emotional demands, workload, and patient care. These conditions often cause physical exhaustion, psychological strain, and reduced job satisfaction. In turn, they negatively affect performance, patient outcomes, and staff retention [1,2]. Addressing these challenges requires an understanding of the factors that drive nurses’ performance, including motivation, WE, and the PSC of the workplace.

### 1.1. Motivation in Nursing Practice

Motivation is a key determinant of performance, retention, and professional commitment among nurses. It can be defined as the internal and external forces that stimulate individuals to initiate, direct, and sustain work-related behaviors [3]. In nursing, motivation encompasses a wide array of influences, including social relationships, organizational culture, communication quality, job satisfaction, work–life balance, and physical working conditions [4,5]. High motivation contributes to higher quality of care, reduced turnover intentions, and stronger organizational loyalty [6].

Theoretical frameworks of work motivation suggest that both intrinsic drivers (e.g., personal growth, professional pride) and extrinsic drivers (e.g., salary, recognition, career advancement) play a role [7]. Research indicates that improvements in the quality of work life, such as providing professional development opportunities, supportive leadership, and adequate staffing, can significantly enhance motivation [8]. Conversely, poor working conditions have been linked to stress, burnout, and higher absenteeism, with burnout reducing nurses’ motivation and increasing their intention to leave the profession [9]. Recent evidence highlights that technology integration and digital health tools can enhance motivation by reducing workload stress through streamlined access to resources and decision-making aids [10]. These patterns underscore the need to examine motivation not in isolation but in relation to other organizational factors.

### 1.2. Work Engagement and Its Relevance to Nursing

WE, closely linked to motivation but conceptually distinct, refers to a positive, fulfilling, work-related state of mind characterized by vigor, dedication, and absorption [11]. Vigor reflects high levels of energy and resilience at work; dedication involves a sense of significance, enthusiasm, and pride; and absorption denotes being fully concentrated and happily engrossed in work tasks.

In nursing, WE is associated with better patient care, higher safety standards, and lower rates of turnover intention [12]. Engaged nurses are more likely to go beyond their job descriptions, collaborate effectively with colleagues, and adapt to changing clinical demands [13]. Factors that enhance engagement include adequate resources, autonomy, supportive supervisors, and positive team dynamics [14]. Beyond workplace conditions, personal and family life also shape engagement. Work–family balance has been shown to improve nurses’ focus and emotional availability, thereby benefiting both employees and patients [15]. Conversely, work–family conflict can drain psychological resources and reduce engagement. Studies from China have demonstrated that psychological flexibility moderates these effects, with more flexible nurses maintaining higher engagement despite stress [16]. Additionally, recent global evidence emphasizes that supportive organizational climates post-COVID-19 are crucial to sustaining engagement in high-demand nursing environments [17].

### 1.3. Psychosocial Safety Climate in Healthcare Settings

The (PSC) is an emerging construct in occupational health psychology that refers to the shared perceptions of organizational policies, practices, and procedures aimed at protecting workers’ psychological health and safety [18]. PSC reflects management’s commitment to mental health, the priority given to psychological safety relative to productivity, the quality of organizational communication about health and safety issues, and the degree of employee participation in decision-making [19].

PSC operates through two primary pathways [20]. The health depletion pathway suggests that negative work conditions, such as high demands, lack of support, and poor communication, erode employee well-being and contribute to burnout. In contrast, the motivational pathway posits that positive work conditions enhance engagement, strengthen morale, and build a healthier work environment. Evidence shows that high PSC is linked to reduced stress, improved morale, and greater organizational commitment [21,22]. In healthcare settings, PSC may be particularly important due to the emotional intensity of the work. Nurses who perceive high PSC are more likely to report feeling safe to express concerns, confident in management’s responsiveness, and committed to their roles. Conversely, low PSC can exacerbate job strain, hinder communication, and contribute to disengagement [23]. A 2025 study further highlights that strengthening PSC in hospitals not only improves staff well-being but also contributes to patient safety and sustainable workforce retention [24].

### 1.4. Interrelationships and the Research Gap

While motivation, WE, and PSC have all been studied in nursing contexts, most research has examined them in isolation. Some studies have shown that PSC predicts WE by fostering supportive environments [21], while others have linked motivation to engagement through mechanisms such as job satisfaction and professional identity [12]. However, little is known about the combined effects of motivation and PSC on engagement, particularly whether PSC influences the relationship between motivation and engagement. This gap is significant for several reasons. First, understanding these dynamics can inform interventions that target both psychological well-being and motivational drivers, leading to stronger and more sustained engagement. Second, most of the evidence comes from high-income countries, while in low- and middle-income settings, such as Egypt, resource limitations, staff shortages, and organizational constraints may reinforce the importance of PSC as a mediating factor. Third, exploring these relationships can help nurse managers design policies that simultaneously address motivation, well-being, and engagement, leading to improved staff retention rates and improved patient outcomes. By addressing this gap, the study seeks to provide evidence-based recommendations for healthcare administrators to improve the work environment, support psychological health, and promote sustained nurse engagement.

### 1.5. Study Aim

This study aims to examine nurses’ perceptions of motivation, PSC, and WE, to explore the relationships among these variables, and to determine whether PSC mediates the association between motivation and engagement.

### 1.6. Research Hypotheses

**Alternative** **Hypothesis** **1.** *Nurses’ motivation is positively associated with WE.*

**Alternative** **Hypothesis** **2.** *Nurses’ motivation is positively associated with PSC.*

**Alternative Hypothesis** **3.** *PSC is positively associated with WE.*

**Alternative** **Hypothesis** **4.** *PSC mediates the relationship between nurses’ motivation and WE.*

## 2. Materials and Methods

Study design: The present study was carried out using a descriptive correlational research design.

Study setting, sample and sampling: The current study was carried out in Assiut University Hospital. A convenience sampling method was employed for this study, including male and female nurses (N = 318), out of 318 participants, 82% were female (*n* = 261) and 18% were male (*n* = 57), with mean age (36.26) ranged as (21.0–56.0) and mean experience (16.27) ranged as (1.0–34.0) working in the predetermined settings. Inclusion criteria: Registered nurses working in direct patient care at Assiut University Hospital with at least one year of clinical experience. Exclusion criteria: Nurses working only in administrative roles, nursing interns, and incomplete questionnaires.

The researchers used “Epi Info 7”. The study utilized a convenience sampling method, incorporating a tool to calculate the required sample size based on the following parameters: a 95% confidence level (Z), and a 5% margin of error (D). This calculation yielded a sample size of 318 participants to ensure a statistical power of 0.80. The sample size estimation was performed using Epi Info, a proprietary software suite developed by the U.S. Centers for Disease Control and Prevention, designed for use by public health professionals and researchers.

Instruments: Socio-demographics: It consists of the data on personal characteristics of the nursing staff. It gathered information such as unit name, age, gender, marital status, educational background, and years of professional experience.

Motivation Scale: This scale was developed by [25], which evaluates the levels of motivation among nurses. It contains 30 items classified into 5 subscales—intrinsic; instrumental; external self-concept; internal self-concept; and goal internalization—with 6 unique statements for every subscale. Every item is represented by a 5-point Likert scale (entirely disagree = 1, somewhat disagree = 2, neutral = 3, somewhat agree = 4, and entirely agree = 5). So, the scoring system ranges from 30 to 150 and it is classified into 3 levels: low motivation among nurses from 30 to 70; moderate motivation among nurses from 71 to 110; high motivation among nurses from 111 to 150.

### 2.1. Psychosocial Safety Climate Scale

Psychosocial Safety Climate Scale: Developed by [23], this scale is designed to assess the psychosocial safety climate within organizations. It consists of 26 items grouped into 4 domains: management support and commitment (10 items), management priority (5 items), organizational communication (6 items), and organizational participation and involvement (5 items).

Scoring System: Responses are rated on a 5-point Likert scale, ranging from 1 (strongly disagree) to 5 (strongly agree). The total score for each domain is calculated and then converted into a percentage. A score of 60% or higher is interpreted as ‘agree,’ while scores below 60% are considered ‘disagree.’ The previous study’s Crombach’s alpha-coefficient value of PSC was 0.77 [24].

### 2.2. Utrecht Work Engagement Scale (UWES)

Work Engagement Scale: Developed by [9], this scale is designed to assess nurses’ levels of work engagement. It comprises 17 items divided into 3 domains: vigor (6 items), dedication (5 items), and absorption (6 items).

Scoring System: Responses are measured using a 3-point Likert scale, with scores ranging from 0 (never), 1 (a few times a month), to 2 (every day), based on how frequently the respondent experiences the described feeling. Total scores range from 0 to 34, with scores between 0 and 17 indicating poor work engagement, and scores between 18 and 34 indicating good work engagement. The previous study‘s Cronbach’s alpha-coefficient value for this tool was 0.82 [26]. Current Cronbach’s α = 0.88.

### 2.3. Validity and Reliability

Tool Validation and Reliability: The final versions of the questionnaires were reviewed by a panel of five experts, three professors, and two assistant professors specializing in nursing administration at Assiut University. The review focused on content relevance, clarity, formatting, length, phrasing, translation into Arabic, and overall presentation. To assess the reliability of the study instruments, Cronbach’s alpha coefficient was calculated. The results demonstrated high internal consistency with values of 0.90 for the Motivation Scale, 0.94 for the Psychosocial Safety Climate (PSC) Scale, and 0.88 for the Utrecht Work Engagement Scale (UWES).

### 2.4. Data Collection

Preliminary Preparation: A preparatory phase was carried out over a period of two months, from January to February 2025. During this time, an extensive review of the relevant national and international literature, both journal articles and academic books, was conducted to support and inform the study’s focus areas.

Pilot Study: A pilot study was conducted to evaluate the clarity, comprehensibility, applicability, and estimated time required for completing the study tools. Additionally, it aimed to identify any potential issues that might arise during the actual data collection process. The pilot study was conducted on 10% of the total sample (*n* = 32). After analyzing the data collected from the pilot, no modifications to the tools were deemed necessary. Therefore, the nurses who participated in the pilot study were excluded from the final sample.

Ethical Approval: Prior to initiating data collection, formal approval was obtained from the hospital’s medical and nursing directors, as well as from the heads of the respective units. Data collection was conducted over a two-month period, from the beginning of Jun to the end of July 2025. Each questionnaire required approximately 15 to 20 min to complete. The study proposal received approval from the Ethical Committee of the Faculty of Nursing.

Participant Interaction and Informed Consent: The researchers met with all participants during their respective shifts, based on their individual schedules. The purpose of the study was explained to each participant, and they were asked for their consent to participate. Following this, the nurses signed an informed consent form and were instructed to complete the questionnaires and return them anonymously.

### 2.5. Data Analysis

Data entry and statistical analysis were conducted using IBM SPSS Statistics, Version 27.0 (IBM Corp., Armonk, NY, USA). Microsoft Excel was employed for data management, and GraphPad Prism 5 was used to create figures.

Prior to analysis, the data were examined for accuracy, missing values, and outliers. The Anderson–Darling test was applied to assess normality. In addition, the assumptions of regression (linearity, homoscedasticity, independence of errors, and absence of multicollinearity) were checked and confirmed to be satisfactory.

Descriptive statistics (frequencies, percentages, means, standard deviations, and ranges) were used to summarize demographic and study variables. Pearson’s product–moment correlation coefficient (two-tailed) with 95% confidence intervals was applied to assess associations among motivation, PSC, and WE.

To identify predictors of the main study outcomes, multivariate linear regression analysis was conducted. Variables were entered based on theoretical relevance, and the final model was determined after examining collinearity and model fit indices.

Mediation analysis was performed to test whether PSC mediated the relationship between motivation and WE. Mediation results were reported with unstandardized coefficients (B), standard errors (SE), *p*-values, and 95% confidence intervals (CI). Effect sizes (proportion of the total effect mediated) were also calculated to provide additional insight into the strength of the mediation pathway. A *p*-value of <0.05 was considered statistically significant for all analyses.

## 3. Results

The study included 318 nurses with a mean age of 36.26 years (SD = 8.97, range = 21–56) and a mean of 16.27 years of professional experience (SD = 8.94, range = 1–34) (see Table 1). The majority were female (82%), married (78%), and held a nursing diploma (59%), while 41% held a bachelor’s degree.

**Figure 1:**
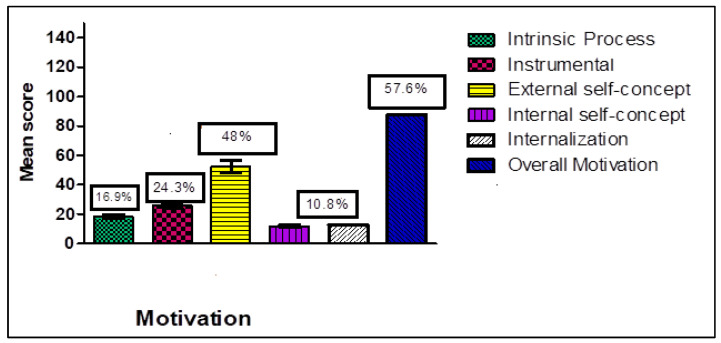
Levels of Motivational Dimensions Among Nurses.

The mean overall motivation score was 57.6% (see Figure 1). Among the motivational dimensions, external self-concept motivation accounted for 48%, instrumental motivation for 24.3%, intrinsic process motivation for 16.9%, and internal self-concept motivation for 10.8%.

**Figure 2:**
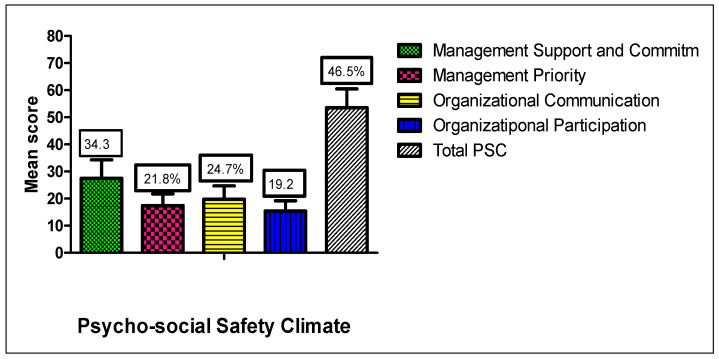
Levels of Psychosocial Safety Climate among Nurses. Note: PSC. Psychosocial Safety Climate.

The mean total PSC score was 46.5% (Figure 2). Management support and commitment scored highest (34.3%), followed by organizational communication (24.7%), management priority (21.8%), and organizational participation (19.2%).

Descriptive statistics (Table 2) showed moderate overall motivation (M = 118.1, SD = 16.4), with external self-concept scoring highest. PSC scores indicated low perceived psychological safety (M = 60.5, SD = 16.6), with management support and commitment rated highest among its dimensions. WE (M = 22.7, SD = 5.8) was also moderate, with vigor as the highest-scoring dimension.

**Figure 3:**
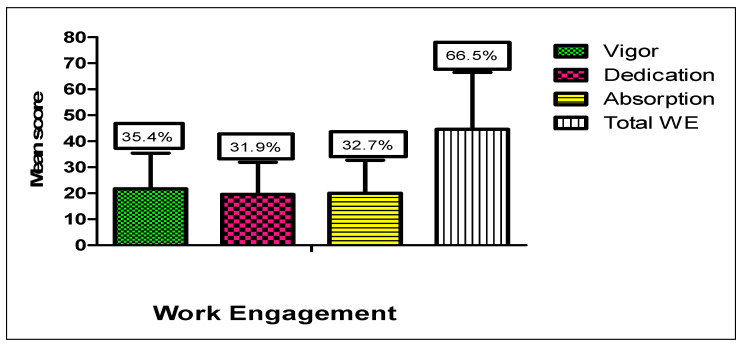
Levels of Work Engagement among Nurses.

Figure 3, showing work engagement dimensions, likely demonstrates moderate levels of vigor, dedication, and absorption among participants. The mean total WE score was 66.5%. Among its dimensions, vigor scored highest (35.4%), followed by absorption (32.7%) and dedication (31.9%).

Correlation analysis revealed a moderate positive association between motivation and PSC (r = 0.48, *p* < 0.001). The relationship between motivation and WE was small and non-significant (r = 0.10, *p* = 0.08). Similarly, PSC and WE were weakly correlated and non-significant (r = 0.09, *p* = 0.09). Educational level correlated positively with PSC (r = 0.21, *p* = 0.035), while years of experience correlated negatively with PSC (r = −0.21, *p* = 0.028) but positively with WE (r = 0.18, *p* = 0.021) (Table 3).

**Figure 4:**
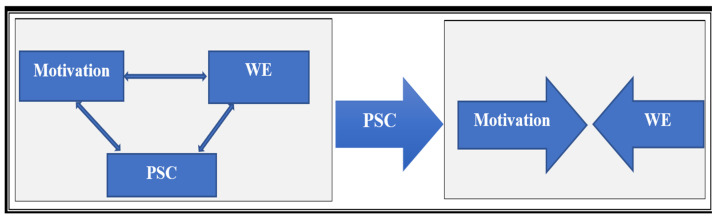
Relationship of mediation between study variables.

A bootstrapped mediation model (5000 resamples) tested whether PSC mediated the relationship between motivation and WE. The direct effect of motivation on WE was small and not statistically significant (B = 0.05, *p* = 0.09). The indirect effect through PSC was statistically significant but small (B = 0.07, 95% CI [0.02, 0.14], *p* = 0.02), indicating partial mediation. The total effect of motivation on WE was significant but modest (B = 0.12, *p* = 0.004) (Table 4, Figure 4).

## 4. Discussion

This study examined the relationships between motivation, PSC, and WE among nurses, as well as the mediating role of PSC. The key findings were (1) nurses reported moderate motivation and WE but low PSC; (2) motivation was moderately associated with PSC, yet both variables showed weak and non-significant associations with WE; and (3) PSC partially mediated the motivation–WE link, but the indirect effect was small.

### 4.1. Motivation and PSC

The moderate, positive correlation between motivation and PSC (r = 0.48) suggests that nurses who perceive stronger psychological safety also tend to be more motivated. This aligns with previous research indicating that motivation and psychological safety are interlinked, and that supportive management, open communication, and participation in decision-making enhance motivation [27,28]. In the present study (Figure 1), external self-concept emerged as the strongest motivational dimension, suggesting that recognition and respect from others are particularly influential in this context. Comparable dynamics were found in ICU nursing settings, where organizational climate fully mediated the effect of transformational leadership on work engagement, highlighting the indispensable role of a positive climate in fostering motivation and commitment [29]. Similar findings have been reported among head nurses in Egypt, where motivation was directly related to stress levels and organizational support structures [30]. However, the low PSC scores observed here indicate that organizational structures for protecting psychological well-being remain insufficient, echoing earlier reports from Egyptian inpatient settings highlighting the need for sustainable nursing practices and improved organizational climates [31,32].

### 4.2. Motivation, PSC, and WE

Contrary to expectations and some previous studies [11,20], neither motivation nor PSC was significantly associated with WE in this study. This finding contrasts with research from Saudi Arabia, where nurses’ personal and job-related factors, including motivation, were strong predictors of WE [33], while longitudinal evidence indicates that PSC boosts engagement and mitigates exhaustion by enhancing job resources and reducing psychological demands [18]. In the present study, the PSC profile (Figure 2) revealed generally low perceptions of psychological safety, with the lowest ratings for participation and organizational engagement. While management support and commitment were the highest-rated PSC domains, they were still well below optimal levels. These gaps suggest that nurses perceive leadership as insufficiently prioritizing psychological well-being and providing limited opportunities for shared decision-making. Similarly, Ref. [34] emphasized that organizational commitment and engagement thrive when psychosocial safety climates are stronger, underscoring the importance of leadership practices.

Also, the WE profile (Figure 3) showed moderate overall engagement, with activity scoring highest, followed by absorption and dedication. This suggests that nurses can maintain energy and focus during their work but may feel less emotionally committed to their roles. The relatively low dedication scores are consistent with PSC findings; when employees feel excluded from decision-making and perceive poor organizational communication, their emotional investment in the job may suffer, even if daily effort remains high. Other contextual explanations include workload, staff shortages, and resource constraints, which have been reported as barriers in Egyptian hospitals during both routine care and COVID-19 pandemic management [35]. Broader systemic issues, such as demographic disparities and healthcare utilization patterns, may also indirectly shape engagement levels [36]. Moreover, while nursing managers often demonstrate creativity supported by rational decision-making [37], in Egypt, cultural expectations and job security perceptions may constrain engagement, consistent with evidence that nurses’ intent to stay is shaped by organizational climate and leadership behaviors [32].

### 4.3. Mediation Role of PSC

Mediation analysis revealed that PSC accounted for a small but significant portion of the relationship between motivation and WE. While statistically significant, the size of the indirect effect (B = 0.07) indicates that PSC alone is insufficient to substantially increase engagement. The Italian study by [21] found that improved PSC significantly reduced relational stressors and emotional exhaustion, indirectly supporting better engagement. Ref. [18] showed PSC’s longitudinal effects in healthcare and education sectors, demonstrating that PSC can enhance engagement and reduce exhaustion through bolstered social support and recognition. Improving PSC may support engagement, but it is not enough on its own. Implementing the educational intervention for head nurses on workplace polarity management was significantly correlated with nurses’ perceptions of their head nurses’ coaching behaviors [38]. Gains are more likely when combined with strategies that reduce workload, increase autonomy, and expand professional growth opportunities. For example, ensuring sustainable nursing activities [31] and building stronger palliative and end-of-life care systems [39] could address systemic stressors and indirectly foster motivation and engagement. Collectively, these findings highlight that organizational and cultural context play an essential role in shaping the motivation–PSC–WE pathway, requiring a holistic approach to workforce sustainability.

### 4.4. Implications for Practice

The findings highlight several practical priorities for nursing leadership that may warrant further research: strengthening PSC by visibly prioritizing psychological health, establishing clear communication channels, and involving nurses in decision-making; enhancing recognition systems that reinforce external self-concept motivation, such as public acknowledgment of achievements and transparent career progression pathways; support intrinsic motivation through mentorship programs, professional development, and opportunities for role expansion; addressing structural barriers to engagement, including staffing adequacy, shift scheduling, and workload management, which may contribute to improved outcomes. These strategies should be further evaluated in future research before being implemented on a large scale.

### 4.5. Strengths and Limitations

The strengths of this study include the use of validated instruments with high reliability and the exploration of mediation, which provides a deeper understanding of the interrelationships between variables. However, the cross-sectional design precludes causal inference. Therefore, the results should be interpreted as correlations, not causal relationships. Future longitudinal or experimental research is needed to confirm the identified mediation pathways, and the use of a convenience sample from a single site limits generalizability. Self-report measures may also be subject to common methodological bias.

### 4.6. Future Research

Future studies should use longitudinal or experimental designs to clarify causal pathways and evaluate the effects of interventions targeting PSC and motivation. Comparative studies across multiple healthcare settings could reveal whether these relationships differ by organizational culture or resource availability.

## 5. Conclusions

This study found that nurses at Assiut University Hospital reported moderate work motivation and engagement but low perceptions of a PSC. Motivation was moderately associated with PSC, and PSC also played a partial role in the relationship between motivation and engagement, although the indirect effect was small. These findings suggest that while supportive organizational climates can enhance motivation, they may not significantly increase engagement on their own without broader workplace improvements. However, given the small effect size and cross-sectional design, these associations should be interpreted with caution. The findings suggest potential directions for nursing leadership, such as promoting a psychosocial safety climate and supporting motivation, that may indirectly enhance engagement and retention. However, further longitudinal and multi-site studies are needed to establish causal relationships and assess the extent to which these strategies can be effectively implemented and sustained in practice.

Future research should employ longitudinal and multi-site designs to establish causal relationships and test targeted interventions. By addressing both psychosocial safety and motivation, healthcare organizations may indirectly foster higher engagement, improve nurse retention, and ultimately enhance patient care quality.

## Figures and Tables

**Table 1:** Demographic Characteristics of the Nursing Staff.

Characteristic	*n*	%	M ± SD	Range
Age (years)			36.26 ± 8.97	21–56
<30	96	30.2		
30–40	114	35.8		
>40	108	34.0		
Educational qualification				
Diploma	189	59.4		
Bachelor’s degree	129	40.6		
Marital status				
Single	69	21.7		
Married	249	78.3		
Years of experience			16.27 ± 8.94	1–34
<10	90	28.3		
10–20	117	36.8		
>20	111	34.9		

Note: *n*, number; %, percentage.

**Table 2:** Descriptive statistics for motivation, PSC, and WE.

Variable/Dimension	M ± SD	Range
Motivation	118.1 ± 16.4	71–186
Intrinsic process	19.9 ± 3.7	8–26
Instrumental	28.7 ± 4.6	20–42
External self-concept	56.6 ± 10.4	31–94
Internal self-concept	17.2 ± 5.0	8–33
Internalization	18.0 ± 3.9	6–25
PSC	60.5 ± 16.6	28–122
Management support & commitment	20.7 ± 7.0	10–46
Management priority	13.1 ± 4.7	5–25
Organizational communication	14.9 ± 4.3	6–30
Participation & involvement	11.6 ± 4.2	5–25
WE	22.7 ± 5.8	8–33
Vigor	8.0 ± 2.1	3–12
Dedication	7.2 ± 2.3	0–10
Absorption	7.4 ± 2.6	0–12

Note: M, mean; SD, standard deviation.

**Table 3:** Significant correlations among key study variables.

Variables	r	95% CI	*p*
Motivation—PSC	0.48	0.39, 0.56	<0.001 *
Motivation—WE	0.10	−0.01, 0.21	0.08
PSC—WE	0.09	−0.02, 0.20	0.09
Education—PSC	0.21	0.01, 0.36	0.035 *
Experience—PSC	−0.21	−0.39, −0.02	0.028 *
Age—WE	0.22	0.03, 0.39	0.021 *

Note: CI, confidence interval; PSC, psycho-social safety climate; WE, work engagement; *p*, probability value; *, statistically significant at *p* < 0.05.

**Table 4:** Mediation analysis of PSC between motivation and work engagement.

Pathway	B	SE	95% CI	*p*
Direct effect (Motivation→WE)	0.05	0.03	−0.01, 0.11	0.09
Indirect effect via PSC	0.07	0.03	0.02, 0.14	0.02 *
Total effect	0.12	0.04	0.04, 0.20	0.004 *

Note: B, unstandardized coefficients; SE, standard errors; CI, confidence intervals; *p*, probability value; *, statistically significant at *p* < 0.05.

## Data Availability

The data presented in this study are openly available.

## Public Involvement Statement

No public involvement in any aspect of this research.

## Guidelines and Standards Statement

This manuscript was drafted against the STROBE (Strengthening the Reporting of Observational Studies in Epidemiology) for observational (descriptive correlational).

## Abbreviations

The following abbreviations are used in this manuscript:

PSC	psychosocial safety climate
WE	work engagement

## References

[1] Jin F., Ni S., Wang L. (2025). Occupational stress, coping strategies, and mental health among clinical nurses in hospitals: A mediation analysis. Front. Public Health.

[2] Dziedzic B., Łodziana K., Marcysiak M., Kryczka T. (2025). Occupational stress and social support among nurses. Front. Public Health.

[3] Kelbiso L., Belay A., Woldie M. (2017). Determinants of motivation among nurses working in Hawassa town public health facilities, South Ethiopia: A cross-sectional study. BMC Nurs..

[4] Kitsios F., Kamariotou M. (2021). Job satisfaction behind motivation: An empirical study in public health workers. Heliyon.

[5] Al-Dossary R.N. (2022). The relationship between nurses’ quality of work-life on organizational loyalty and job performance in Saudi Arabian hospitals: A cross-sectional study. Front. Public Health.

[6] Zaghini F., Fiorini J., Piredda M., Fida R., Sili A. (2020). The relationship between nurse managers’ leadership style and patients’ perception of the quality of the care provided by nurses: A cross-sectional survey. Int. J. Nurs. Stud..

[7] Ruzevicius J., Valiukaite J. (2017). Quality of life and motivation balance: Case study of public and private sectors of Lithuania. Qual. Access Success.

[8] Azevedo B.D.S., Nery A.A., Cardoso J.P. (2017). Occupational stress and dissatisfaction with motivation in nursing. Texto. Contexto. Enferm..

[9] Azzellino G., Dante A., Petrucci C., Caponnetto V., Aitella E., Lancia L., Ginaldi L., De Martinis M. (2025). Intention to leave and missed nursing care: A scoping review. Int. J. Nurs. Stud. Adv..

[10] Awosiku O.V., Gbemisola I.N., Oyediran O.T., Egbewande O.M., Lami J.H., Afolabi D., Okereke M., Effiong F. (2025). Role of digital health technologies in improving health financing and universal health coverage in Sub-Saharan Africa: A comprehensive narrative review. Front. Digit. Health.

[11] Rastogi M., Saikia A. (2019). Determinants of work engagement among nurses in Northeast India. J. Health Manag..

[12] Kim E., Lee J.Y., Lee S.E. (2023). Associations among leadership, resources, and nurses’ work engagement: Findings from the fifth korean Working Conditions Survey. BMC Nurs..

[13] Wang Y., Gao Y., Xun Y. (2021). Work engagement and associated factors among dental nurses in China. BMC Oral Health.

[14] Platania S., Morando M., Caruso A., Scuderi V. (2022). The effect of psychosocial safety climate on engagement and psychological distress: A multilevel study on the healthcare sector. Safety.

[15] Zinsser K., Zinsser A. (2016). Two case studies of preschool psychosocial safety climates. Res. Hum. Dev..

[16] Dollard M., Dormann C., Idris M.A. (2019). Psychosocial safety climate: A new work stress theory and implications for method. Psychosocial Safety Climate.

[17] Bal P.M., Dóci E. (2018). Neoliberal ideology in work and organizational psychology. Eur. J. Work Organ. Psychol..

[18] Bourgoin Boucher K., Ivers H., Biron C. (2024). Mechanisms explaining the longitudinal effect of psychosocial safety climate on work engagement and emotional exhaustion among education and healthcare professionals during the COVID-19 pandemic. Int. J. Environ. Res. Public Health.

[19] Bond S., Tuckey M., Dollard M. (2010). Psychosocial safety climate, workplace bullying, and symptoms of posttraumatic stress. Organ. Dev. J..

[20] Garrick A., Mak A., Cathcart S., Winwood P., Bakker A., Lushington K. (2014). Psychosocial safety climate moderating the effects of daily job demands and recovery on fatigue and work engagement. J. Occup. Health Psychol..

[21] Galanti T., Cortini M., Giudice G.F., Zappalà S., Toscano F. (2024). Safeguarding nurses’ mental health: The critical role of psychosocial safety climate in mitigating relational stressors and exhaustion. AIMS Public Health.

[22] Bakertzis E., Myloni B. (2021). Profession as a major drive of work engagement and its effects on job performance among healthcare employees in Greece: A comparative analysis among doctors, nurses and administrative staff. Health Serv. Manag. Res..

[23] Dollard M., Kang S. (2007). Psychosocial Safety Climate Measure.

[24] Akanni A., Ajila C., Omisile I., Ndubueze K. (2021). Mediating effect of work self-efficacy on the relationship between psychosocial safety climate and workplace safety behaviors among bank employees after COVID-19 lockdown. Cent. Eur. Manag. J..

[25] Barbuto J.E., Scholl R.W. (1998). Motivation sources inventory: Development and validation of new scales to measure an integrative taxonomy of motivation. Psychol. Rep..

[26] Ali H. (2018). Head nurses’ interpersonal relationship and its effect on work engagement and proactive work behavior at Assuit University Hospitals [master’s thesis]. Assiut Sci. Nurs. J..

[27] Abu-Qutaish R., Alosta M.R., Abu-Shosha G., Oweidat I.A., Nashwan A.J. (2025). The relationship between transformational leadership, work motivation, and engagement among nurses in Jordanian governmental hospitals. BMC Nurs..

[28] Qiu Y., Yang Y., Wang J., Wang Q., Zhao S., Ding X. (2025). The influence of self-determined motivation on patient safety competency among nurses: The chain mediating effect of psychological contract and psychological capital. Nurse Educ. Today.

[29] Zhang L., Han L., Liang X., Wang R., Fan H., Jia Y., Li S., Jiang X. (2025). The relationship between transformational leadership and work engagement among intensive care unit nurses: The mediating function of organizational climate. BMC Nurs..

[30] Ahmed G., Abdelazeem S., Abdallah H. (2020). The relationship between motivation and occupational stress among head nurses in Port Said hospitals. Port Said Sci. J. Nurs..

[31] Hossny E.K. (2022). Studying nursing activities in inpatient units: A road to sustainability for hospitals. BMC Nurs..

[32] Hossny E.K., Alotaibi H.S., Mahmoud A.M., Elcokany N.M., Seweid M.M., Aldhafeeri N.A., Abdelkader A.M., Elhamed S.M.A. (2023). Influence of nurses’ perception of organizational climate and toxic leadership behaviors on intent to stay: A descriptive comparative study. Int. J. Nurs. Stud. Adv..

[33] Alkorashy H., Alanazi M. (2023). Personal and job-related factors influencing the work engagement of hospital nurses: A cross-sectional study from Saudi Arabia. Healthcare.

[34] Lateef S.F., Mohamed F.R., Hossny E.K. (2021). The relationship between motivation, psycho-social safety climate and nursing staff work engagement and organizational commitment. Assiut Sci. Nurs. J..

[35] Hossny E.K., Morsy S.M., Ahmed A.M., Saleh M.S.M., Alenezi A., Sorour M.S. (2022). Management of the COVID-19 pandemic: Challenges, practices, and organizational support. BMC Nurs..

[36] Mohamed A.M. (2023). The effect of COVID-19 on health insurance: The mediating role of demographics and healthcare service utilization. Sci. J. Commer. Res..

[37] Hossny E.K., Alotaibi H.S. (2024). Relationship between dominant decision-making style and creativity of nursing managers: A cross-sectional study. BMC Nurs..

[38] Sorour M.S., Hossny E.K., Mohamed N.T., Abdelkader A.M., Alotaibi H.S., Obied H.K. (2023). Effect of head nurses’ workplace polarity management educational intervention on their coaching behavior. Nurs. Res. Pract..

[39] Eltaybani S., Igarashi A., Yamamoto-Mitani N. (2020). Palliative and end-of-life care in Egypt: Overview and recommendations for improvement. Int. J. Palliat. Nurs..

